# Extracellular Release of Citrullinated Vimentin Directly Acts on Osteoclasts to Promote Bone Resorption in a Mouse Model of Periodontitis

**DOI:** 10.3390/cells12081109

**Published:** 2023-04-08

**Authors:** Satoru Shindo, Roodelyne Pierrelus, Atsushi Ikeda, Shin Nakamura, Alireza Heidari, Maria Rita Pastore, Elizabeth Leon, Sunniva Ruiz, Harsh Chheda, Rhea Khatiwala, Tomoki Kumagai, George Tolson, Islam Elderbashy, Kazuhisa Ouhara, Xiaozhe Han, Maria Hernandez, Saynur Vardar-Sengul, Hideki Shiba, Toshihisa Kawai

**Affiliations:** 1 Department of Oral Science and Translational Research, College of Dental Medicine, Nova Southeastern University, 3200 South University Drive, Fort Lauderdale, FL 33328, USA; 2 Department of Periodontics and Endodontics, Okayama University Hospital, 2-5-1 Shikata-cho, Kita-ku, Okayama 700-8525, Japan; 3 Department of Biological Endodontics, Graduate School of Biomedical and Health Sciences, Hiroshima University, 1-2-3 Kasumi, Minami-ku, Hiroshima 734-8553, Japan; 4 Department of Periodontology, College of Dental Medicine, Nova Southeastern University, 3200 South University Drive, Fort Lauderdale, FL 33328, USA; 5 Department of Periodontal Medicine, Graduate School of Biomedical and Health Sciences, Hiroshima University, 1-2-3 Kasumi, Minami-ku, Hiroshima 734-8553, Japan

**Keywords:** citrullinated vimentin, osteoclast, periodontitis, Cl-amidine, PKC-δ, peptidyl arginine deiminase

## Abstract

Elevated osteoclast (OC)-mediated bone resorption, a common pathological feature between periodontitis and rheumatoid arthritis (RA), implicates a possible mutually shared pathogenesis. The autoantibody to citrullinated vimentin (CV), a representative biomarker of RA, is reported to promote osteoclastogenesis (OC-genesis). However, its effect on OC-genesis in the context of periodontitis remains to be elucidated. In an in vitro experiment, the addition of exogenous CV upregulated the development of Tartrate-resistant acid phosphatase (TRAP)-positive multinuclear OCs from mouse bone marrow cells and increased the formation of resorption pits. However, Cl-amidine, an irreversible pan-peptidyl arginine deiminase (PAD) inhibitor, suppressed the production and secretion of CV from RANKL-stimulated OC precursors, suggesting that the citrullination of vimentin occurs in OC precursors. On the other hand, the anti-vimentin neutralizing antibody suppressed in vitro Receptor activator of nuclear factor kappa-Β ligand (RANKL)-induced OC-genesis. The CV-induced upregulation of OC-genesis was abrogated by the Protein kinase C (PKC)-δ inhibitor Rottlerin, accompanied by the downmodulation of OC-genesis-related genes, including Osteoclast stimulatory transmembrane protein (OC-STAMP), TRAP and Matrix Metallopeptidase 9 (MMP9) as well as extracellular signal-regulated kinase (ERK) mitogen-activated protein (MAP)-kinase phosphorylation. Elevated levels of soluble CV and vimentin-bearing mononuclear cells were found in the bone resorption lesions of periodontitis induced in mice in the absence of an anti-CV antibody. Finally, local injection of anti-vimentin neutralizing antibody suppressed the periodontal bone loss induced in mice. Collectively, these results indicated that the extracellular release of CV promoted OC-genesis and bone resorption in periodontitis.

## 1. Introduction

It is well-established that periodontitis is induced by a host immune response to pathogenic factors produced by opportunistic pathogens in the dental plaque, which, in turn, leads to tissue-destructive inflammation of the periodontium, including alveolar bone [1,2,3].

Osteoclasts (OCs) are responsible for bone resorption in inflammatory bone lytic diseases such as rheumatoid arthritis (RA) [4,5] and periodontitis [6,7]. OC-genesis, the process of differentiating into multinucleated OCs from their mononuclear precursors, is elicited by the receptor activator of the nuclear factor-kappa B (NF-κB) ligand (RANKL) in the presence of permissive macrophage colony-stimulating factor (M-CSF) [8,9]. RANKL is locally produced by osteoblasts, osteocytes and periodontal ligament cells, all of which originate from mesenchymal stem cells [10,11,12,13]. Lymphocytes, including T cells and B cells, are also a relevant source of RANKL in periodontitis [14,15,16]. The consensus supports that locally produced proinflammatory factors such as Interleukin-1β, Tumor necrosis factor-α and Prostaglandin E2 contribute to elevated OC-genesis in periodontitis [17,18]. However, the evidence also suggests that proinflammatory cytokines do not necessarily promote periodontal bone loss. To explain, the gingival inflammation found in gingivitis does not always cause periodontal bone loss [19]. Furthermore, cases of non-inflammatory destructive periodontal disease have been reported to cause massive bone loss [20]. Thus, it can be concluded that the molecular and cellular mechanisms underlying pathologically elevated OC-genesis in periodontitis remain elusive and that other, still unknown, factors may be responsible for promoting OC-genesis in periodontitis.

RA is a chronic inflammatory lesion in the synovial tissue of multiple joints caused by autoimmune responses, characterized by elevated autoantibodies to self-antigens such as collagen and vimentin [21]. Based on the common pathologic manifestations of periodontitis and RA, including inflammatory bone resorption, mutually shared etiopathogenic agents between these diseases have been sought [22,23]. A distinctively elevated anti-citrullinated protein antibody (ACPA) has been detected in the serum of RA patients [24]. Among several autoantibodies detected in RA patients, anti-cyclic citrullinated peptide (CCP) antibodies are specific markers of RA [24,25]. On the other hand, autoantibodies to mutated citrullinated vimentin (MCV) are also known to be highly sensitive markers for RA [26,27,28,29]. For these reasons, the combination of anti-CCP and anti-MCV is considered to increase both sensitivity and specificity for the diagnosis of RA [30,31].

Citrullinated vimentin (CV) and MCV share common autoantibodies that react to citrullinated autoantigens. Citrullination is mediated by peptidyl arginine deaminase (PAD) enzymes, which metabolize the amino acid arginine in a protein into citrulline [32]. Vimentin, which is a specific cytoskeletal protein of mesenchymal and hematopoietic cells, is citrullinated by PAD in the inflammatory lesion of rheumatic diseases, including RA [33,34]. CV is found in the synovium of patients with RA [34,35]. Furthermore, the anti-MCV antibody isolated from human RA patients was reported to promote OC-genesis from human osteoclasts by acting on CV produced by OC precursors [36]. On the other hand, the anti-MCV antibody induced periarticular bone loss and OC-genesis in a mouse model of RA [37]. An elevated level of vimentin has been reported in the gingival crevice fluid (GCF) of patients with periodontitis [38]. Interestingly, these patients showed significantly higher titers of the anti-vimentin antibody, but not the anti-MCV antibody [39], suggesting that the above-noted anti-MCV antibody-mediated promotion of OC-genesis [36] may not occur in periodontitis. Thus, it is largely unknown whether CV has any impact on OC-genesis in the absence of the anti-MCV antibody. To address these unknowns in the present study, we examined the effects of CV on in vitro OC-genesis and periodontal bone loss induced in a mouse model of periodontitis.

## 2. Materials and Methods

### 2.1. Animals

C57BL/6 mice (8 to 10 weeks old) were kept in a conventional room with a 12 h light–dark cycle at a constant temperature. The experimental procedures employed in this study were approved by the Forsyth IACUC (Protocol #14-015) and NSU IACUC (Protocol #TK6).

### 2.2. Cell Culture

To obtain osteoclast precursor cells, bone marrow mononuclear cells (BMMCs) were collected from tibias and femurs of C57BL/6 mice and were cultured in the wells of a 96-well plate (3.0 × 10^5^ cells/well) or 24-well plate (1.0 × 10^6^ cells/well) with a minimum essential medium-α (MEM-α) supplemented with 10% fetal bovine serum (FBS), 100 µg/mL streptomycin and 100 U/mL penicillin as well as 292 μg/mL L-glutamine and 50 ng/mL M-CSF (BioLegend) at 37 °C in humidified air with 5% CO_2_ for 3 days. Subsequently, the OC precursors were differentiated to osteoclasts using 50 ng/mL RANKL (BioLegend, San Diego, CA, USA).

### 2.3. Citrullination of Vimentin

Recombinant human vimentin (PeproTech, Cranbury, NJ, USA) or bovine serum albumin (BSA) (Sigma-Aldrich, St. Louis, MO, USA) was incubated with 400 mU/mL recombinant human PAD2 (Sigma-Aldrich) in the presence of 100 mM Tris-HCl (pH 7.6), 10 mM CaCl_2_ and 5 mM dithiothreitol at 37 °C for 24 h according to Ishigami et al. [40]. 

### 2.4. Enzyme-Linked Immunosorbent Assay (ELISA) for Detection of CV

An anti-CV ELISA was developed using an anti-citrullinated protein antibody (rabbit polyclonal, Abcam, Waltham, MA, USA: capture Ab) and anti-vimentin mAb (clone: 3CB2, mouse IgM, Iowa Hybridoma Bank, Iowa City, IA, USA: detection Ab). As a capture antibody, 2 μg/mL of an anti-citrullinated protein antibody was added to each well of a High Binding 96-well plate (Corning, Corning, NY, USA) at room temperature (RT) overnight. After blocking with 1% BSA in phosphate-buffered saline with 0.05% Tween-20 (PBS-T) at RT for 30 min, the wells were incubated with CV, citrullinated BSA or gingival crevicular fluid (GCF) samples at RT for 1 h. As a detection antibody, 2 μg/mL of anti-vimentin mAb was added to each well and incubated at RT for 1 h. After washing with PBS-T, 1:2000 diluted horseradish peroxidase (HRP)-conjugated anti-mouse IgM antibody or HRP-conjugated anti-mouse IgG antibody (Jackson Laboratory, Bar Harbor, ME, USA) was applied to the respective wells and incubated at RT for 1 h. The color development of the substrate, *o*-Phenylenediamine dihydrochloride (Sigma-Aldrich), reacted to the peroxidase and was measured at a 450 nm spectrum using a microplate reader (Synergy H, BioTek, Santa Clara, CA, USA).

### 2.5. Tartrate-Resistant Acid Phosphatase (TRAP) Staining

OC precursors were stimulated by RANKL with or without vimentin, CV, Rottlerin (Protein kinase C (PKC)-δ inhibitor; Thermo Fisher Scientific, Waltham, MA, USA), KT 5720 (PKA inhibitor, Santa Cruz Biotechnology, Dallas, TX, USA), anti-vimentin mAb (mouse IgM, clone 3CB2) or isotype control mAb (mouse IgM, clone TEPC183, Ancell Corp., Stillwater, MN, USA) for 7 days with the addition of a fresh medium change every 3 days. TRAP staining was performed with a TRAP staining kit (Sigma-Aldrich) according to the manufacturer’s protocol. Briefly, the cells were fixed by a citrate (0.38 mol/L)/acetone solution for 30 s at room temperature. After washing with deionized water, the cells were stained with a TRAP staining solution (L(+)-tartrate buffer, 0.67 mol/L; acetate buffer, 2.5 mol/L; Naphthol AS-BI phosphoric acid, 12.5 mg/mL; and Fast Garnet GBC base, 7.0 mg/mL ) for 10 min at 37 °C in the dark. Cells with ≥ 3 nuclei were determined as multinucleated OCs.

### 2.6. Pit Formation Assay

A plate coated with calcium phosphate was prepared according to previous reports [41,42]. Briefly, 0.12 M Na_2_HPO_4_ and 0.2 M CaCl_2_ (50 mM Tris-HCl, pH 7.4) were mixed at 37 °C. The calcium phosphate slurry was washed with sterile water and then applied into wells of a 96-well plate and dried at 37 °C overnight. BMMCs (3.0 × 10^5^ cells/well) were then seeded in the calcium phosphate-coated plate. After the OC differentiation, the plates were washed with 10% sodium hypochlorite for 10 min and then dried overnight. The pit areas were microscopically imaged (Evos Cell Imaging System, Thermo Fisher Scientific). The image analysis was carried out using ImageJ software (version 1.50).

### 2.7. Measurement of C-Terminal Telopeptide of Type I Collagen (CTX-1) 

A 96-well tissue culture plate, the surface of which was attached to hydroxyapatite, was further coated with type I collagen (50 μg/mL, MD Bioproducts, Oakdale, MN, USA) by incubation at room temperature for one hour. In a few experiments, calvaria bone disks freshly dissected from mice using a biopsy punch (Φ 6 mm) were placed into a plain uncoated 96-well plate. OC precursors were applied to the pretreated plate and stimulated with M-CSF and RANKL in the presence or absence of anti-vimentin mAb or isotype control mAb for 7 days. During the 7 day culture, 50% of the culture medium was exchanged for a fresh medium with all necessary additives every 3 days. The supernatant collected on day 7 was subjected to a CTX-1 ELISA (Novus Biologicals, Centennial, CO, USA). For the in vivo experiment, the blood serum collected from mice induced with periodontitis was subjected to a CTX-1 ELISA (10- and 100-fold dilution).

### 2.8. Quantitative Polymerase Chain Reaction (qPCR)

Total RNA was extracted from OC precursors using an RNA extraction kit (Thermo Fisher Scientific), following the manufacturer’s protocol. First strand cDNA was assembled from 100 ng of sample RNA using a Verso cDNA Synthesis Kit (Thermo Fisher Scientific). Amplification reactions were performed by Taqman Fast Advanced Master Mix (Thermo Fisher Scientific). Polymerase activation was employed for 20 s at 95 °C. PCR was performed with 40 cycles of 1 s at 95 °C and 20 s at 60 °C. The resultant cDNA was amplified by specific probes (Thermo Fisher Scientific) for Gapdh (Mm99999915_g1), Ocstamp (Mm00512445_m1), Mmp9 (Mm00442991_m1), Ctsk (Mm00484039_m1), Acp5 (Mm00475698_m1) and Efnb2 (Mm00438670_m1) on a QuantStudio™ 3 (Thermo Fisher Scientific). The ratios of mRNA levels to those of the control gene were calculated using the ΔCt method (2^−ΔΔCt^). 

### 2.9. PCR Array

After the extraction of total RNA from the cells, reverse transcription was performed using an RT^2^ First Strand Kit (QIAGEN, Germantown, MD) and the resultant cDNA was tested using an RT^2^ Profiler™ PCR Array Mouse GPCR Signaling Pathway Finder (PAMM-071ZF, QIAGEN) according to the manufacturer’s protocol. An integrated web-based software package was used for the data analysis (www.SABiosciences.com/pcrarraydataanalysis.php: accessed on 3 March 2017).

### 2.10. Western Blot

To determine the possible effect of CV on cell signaling induced in RANKL-primed OC precursors, a Western blot (W-blot) analysis was performed to detect the phosphorylation of the following cell signaling molecules: PKC-δ, p38 mitogen-activated protein kinases (p38 MAPK), extracellular signal-regulated kinase (ERK) and c-Jun N-terminal kinase (JNK). The citrullination of vimentin occurring in the OC precursors was also determined using W-blot. OC precursors primed with M-CSF were stimulated with or without RANKL and/or CV in the presence or absence of Rottlerin or Cl-amidine (pan-PAD inhibitor; Cayman Chemical, Ann Arbor, Michigan). After incubations for various periods, the OC precursors were lysed by incubation on ice for 30 min with a RIPA buffer (1 M Tris-HCl pH: 7.8, 2.5 M NaCl, 0.5 M EDTA pH: 8.0, 10% NP-40, 2.5% Na-deoxicholate and 20% SDS) supplemented with a protease inhibitor cocktail (Sigma-Aldrich). The protein concentration of the resultant lysates was measured with a BCA Protein Assay Kit (Thermo Fisher Scientific). A total of 15 μg of sample per lane was loaded onto a 4–12% sodium dodecyl sulfate–polyacrylamide gel electrophoresis (SDS-PAGE) gel (Thermo Fisher Scientific). The proteins separated in the SDS-PAGE gel were electro-transferred to a polyvinylidene difluoride membrane. The detection of specific proteins in the OC precursors was assessed using anti-phospho-PKC-δ rabbit mAb (1:1000; Cell Signaling Technology, Danvers, MA), anti-PKC-δ rabbit mAb (1:1000; Cell Signaling Technology), anti-phospho-p38 MAPKs rabbit mAb (1:1000; Cell Signaling Technology), anti-p38 MAPKs rabbit mAb (1:1000; Cell Signaling Technology), anti-phospho-ERK rabbit mAb (1:2000; Cell Signaling Technology), anti-ERK rabbit mAb (1:2000; Cell Signaling Technology), anti-phospho-JNK rabbit mAb (1:1000; Cell Signaling Technology), anti-JNK rabbit mAb (1:1000; Cell Signaling Technology), biotin-conjugated vimentin rabbit mAb (1:1000; Sigma-Aldrich), anti-citrulline rabbit mAb (1:500; Abcam) or an anti-β-actin rabbit polyclonal antibody (Cell Signaling Technology). Protein bands that reacted with the respective antibody were visualized by incubation with an HRP-conjugated rabbit secondary antibody (Jackson ImmunoResearch, West Grove, PA) or HRP-conjugated streptavidin (Thermo Fisher Scientific), followed by detection using a Luminata Forte Western HRP Substrate (Sigma-Aldrich). The densitometric analysis was performed using ImageJ software (version 1.50)

### 2.11. cAMP Assay

The cAMP extraction from the OC precursors and the measurement of cellular cAMP levels was performed using a Cyclic AMP EIA Kit (Cayman Chemical) according to the manufacturer’s instructions.

### 2.12. Periodontal Disease Model

For the induction of periodontal disease in C57BL6/J mice (6–8 weeks old; male *n* = 5/group), the maxillary second molar was ligatured with 5-0 silk, following the previously published protocol [43,44]. An anti-vimentin monoclonal antibody (5 μg/site, clone: 3CB2, mouse IgM) or isotype control IgM mAb (5 μg/site, clone: TEPC183, mouse IgM) was locally injected into the gingival tissue of a ligature-attached site every 2 days (day 0, 2, 4 and 6). After 7 days, the mice were euthanized for postmortem analyses (Section 2.13, Section 2.14 and Section 2.15).

### 2.13. Measurement of Alveolar Bone Resorption

Resected maxillae were mechanically removed and exposed to 3% hydrogen peroxide overnight to remove all soft tissue. To evaluate the periodontal bone resorption, the distances from the cement–enamel junction to the alveolar bone crest on the buccal side of each root were measured for the maxillary second molar under the microscope, following the previously published protocol [43,44].

### 2.14. Measurement of Vimentin and CV in Mouse GCF

Murine GCF sampling methods followed a previous report [3]. Briefly, after the induction of periodontitis with a silk ligature by the above method, the old ligature was removed and new fresh ligature was mounted around the tooth for 10 min to collect the GCF. The amount of vimentin and CV in these samples was measured by an ELISA.

### 2.15. Histological Analysis

Murine maxillary bones were fixed in 4% paraformaldehyde overnight at 4 °C before decalcification in 10% EDTA at 4 °C for 2 weeks. The tissues were embedded in an OCT compound (Sakura Finetek USA, Torrance, CA, USA) overnight at −20 °C and cut into 8 μm sections with a cryostat. For the immunohistochemistry assessment, the endogenous peroxidase activity was neutralized by 3% H_2_O_2_ for 10 min. After blocking with 5% BSA in PBS, the sections were incubated with biotin-conjugated anti-vimentin mAb (Sigma-Aldrich) overnight at 4 °C, followed by incubation with an avidin–biotin complex containing horseradish peroxidase (ABC kit, Vector Laboratories, Burlingame, CA, USA). Specific staining was performed with a DAB substrate (Vector Laboratories). TRAP staining of the decalcified periodontal tissue was performed using an Acid Phosphatase Leukocyte (TRAP) Kit (Sigma-Aldrich), described in Materials and Methods Section 2.5, followed by nuclear counterstaining with methyl green. The sections were imaged with an EVOS XL Core (Thermo Fisher Scientific). For the immunofluorescence-based detection of OC-STAMP and phospho-PKC-δ, the sections were reacted with anti-OC-STAMP rabbit pAb (1:200; Sigma-Aldrich) or anti-phospho-PKC-δ rabbit mAb (1:200; Cell Signaling Technology) as the primary antibody at 4 °C overnight. Cy3-conjugated anti-rabbit IgG FC goat pAb (1:200; Jackson ImmunoResearch) was used as a secondary antibody. The stained sections were mounted with Fluoromount-G containing DAPI (Thermo Fisher Scientific). The immunofluorescence was observed with a Zeiss LSM880 Confocal Microscope (Carl Zeiss, Jena, Germany).

### 2.16. Statistical Analysis

Statistical analyses were performed by a one-way ANOVA and Tukey HSD to compare the differences between multiple groups and a Student’s *t*-test for comparisons between two groups. A statistical significance was considered to be at *p* < 0.05. All data were expressed as the mean ± SD.

## 3. Results

### 3.1. CV Promotes Osteoclast Differentiation

**Figure 1:**
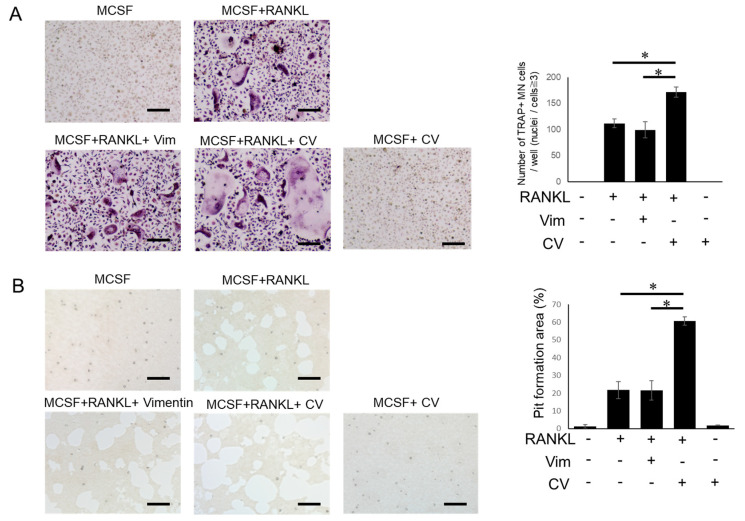
CV promotes RANKL-mediated OC-genesis. M-CSF (20 ng/mL)-primed OC precursors derived from mouse bone marrow were stimulated with RANKL (50 ng/mL) in the presence or absence of vimentin (Vim) or CV for 7 days by exchanging 50% of culture medium with fresh medium every 3 days. (**A**) TRAP staining and (**B**) pit formation assay were performed. The number of TRAP-positive cells with more than 3 nuclei were counted. The resorption pit area was measured using ImageJ. All in vitro assays were performed in triplicate and the results were expressed by the mean ± SD. All experiments were independently performed three times. * *p* < 0.01. Scale bar: 100 μm.

In order to examine the effect of vimentin and citrullinated vimentin (CV) on osteoclastogenesis (OC-genesis), M-CSF-primed OC precursors (bone marrow cells from C57BL6 mice) were stimulated with RANKL in the presence or absence of vimentin or CV. Both multinuclear TRAP-positive osteoclasts (Figure 1A) and resorption pits (Figure 1B) were promoted by the addition of CV, but there was little or no induction by vimentin, suggesting that CV could promote not only osteoclastogenesis, but also the bone resorption activity of differentiated osteoclasts.

### 3.2. PAD-Dependent Citrullinated Vimentin Produced by OC Precursors Is Engaged in the Promotion of RANKL-Induced Osteoclastogenesis

**Figure 2:**
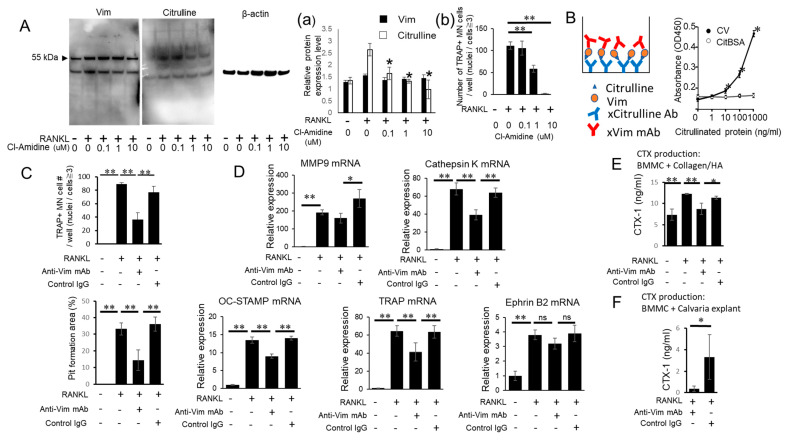
Possible engagement of Cl-amidine-sensitive PAD in the citrullination of vimentin and anti-vimentin mAb-mediated interruption of CV-induced OC-genesis. (**A**) OC precursors were stimulated with or without RANKL (50 ng/mL) and/or Cl-amidine. After 24 h, supernatant and cell lysates were collected and vimentin (Vim) or citrulline was detected by Western blotting using the antibodies described in the Materials and Methods section. Protein bands were measured by densitometry to determine the levels of Vim, citrulline and β-actin and relative expression of Vim and citrulline was calculated in comparison with an internal control of β-actin (Figure 2(Aa)). After 6 days of above-noted OC-genesis conditions, TRAP staining was performed (Figure 2(Ab)). (**B**) Sandwich ELISA to determine citrullination of Vim. Anti-citrulline antibody was used as a capture antibody and anti-Vim antibody as a detection antibody. Different concentrations of CV and citrullinated BSA (CitBSA) (0, 1, 10, 100 and 1000 ng/mL) were used as a sample. (**C**,**D**) OC precursors were stimulated with RANKL in the presence or absence of anti-Vim mAb or control IgG for 48 h or 7 days with the addition of fresh medium every 3 days. TRAP staining, pit formation assays and qPCR were performed. (**E**,**F**) OC precursors were cultured on a type I collagen-treated hydroxyapatite coating plate (**E**) or on murine calvaria bone disks (**F**). After 7 days, the supernatant was applied to CTX-1 ELISA. All in vitro assays were performed in triplicate and the results were expressed as the mean ± SD. All experiments were independently repeated three times. * *p* < 0.05; ** *p* < 0.01. #: number.

The anti-MCV autoantibody isolated from human patients with RA has been reported to promote OC-genesis by acting on CV produced by OC precursors [36]. However, the biological property of CV in modulating OC-genesis in the absence of the anti-MCV autoantibody is not known. This is important to the present study and in periodontitis without rheumatoid arthritis, although the level of vimentin is elevated in gingival crevice fluid [38]. However, in the context of the present study, it is important to note that anti-MCV autoantibody levels are not increased in patients with periodontitis without rheumatoid arthritis [39]. In accordance with the report by Harre et al. [36], the OC precursors stimulated with RANKL produced CV (Figure 2A; Western blot), but such a production was inhibited by Cl-amidine, a chemical inhibitor of PAD, although it did not affect the total vimentin (Figure 2(Aa)). These results indicated that the citrullination of vimentin in the OC precursors was mediated by PAD and that Cl-amidine could suppress the citrullination of vimentin without affecting its expression level. Furthermore, an additional OC-genesis assay demonstrated that Cl-amidine significantly suppressed RANKL-mediated osteoclastogenesis (Figure 2(Ab)), suggesting that CV could promote OC-genesis. 

### 3.3. Anti-Vimentin mAb-Mediated Blocking Can Inhibit the Pro-OC-Genesis Effect by CV

To investigate whether CV expressed by RANKL-primed OC precursors was engaged in OC-genesis, anti-vimentin mAb was employed to block the activity of CV. To this end, we first confirmed the binding activity of anti-vimentin mAb to CV (Figure 2B) using a sandwich ELISA. Anti-citrulline mAb coated on the ELISA plate captured both CV and citrullinated BSA. Anti-vimentin mAb applied as the secondary antibody then reacted with CV, but not citrullinated BSA (Figure 2B), suggesting that anti-vimentin mAb bound to the non-citrullinated epitope present in CV.

In the in vitro RANKL-induced osteoclastogenesis assay, the addition of anti-vimentin mAb, but not control mAb, significantly suppressed the differentiation of TRAP+ multinuclear OC cells from the OC precursors stimulated with RANKL (Figure 2C) as well as the genes associated with OC-genesis such as *Mmp9*, *Acp5*, *CatK* and *Ocstamp*. EphrinB2 expressed by OCs and EphB4 expressed by OBs were recently identified as key coupling factors, whose interaction stimulates bone formation [45]. However, at a gene level, EphrinB2 mRNA was not significantly affected by the treatment with anti-vimentin mAb (Figure 2D). The level of resorption pit formation mediated by RANKL-stimulated osteoclasts was also suppressed by the addition of anti-vimentin mAb, whereas control mAb did not affect the activity of osteoclasts in generating resorption pits (Figure 2C). In the course of bone resorption, it is well-established that osteoclasts not only resorb hydroxyapatite, but also degrade proteins present in the bone [46,47]. The osteoclast-mediated degradation of type I collagen, one of the most abundant proteins in bone, can generate C-terminal telopeptide (CTX-1), which is accepted as a serum biomarker for osteoporosis and rheumatoid arthritis [48,49] as well as a salivary biomarker for periodontitis [50,51]. As expected, the culture of RANKL-stimulated OC precursors on the type I collagen-coated hydroxyapatite plate and on calvarial bone disks increased the CTX-1 concentration in the culture supernatant (Figure 2E,F). In both cases, the addition of anti-vimentin mAb, but not control mAb, significantly suppressed the release of CTX-1 into the culture supernatant (Figure 2E,F). These results indicated that CV produced by the OC precursors in an autocrine fashion was engaged in the promotion of RANKL-induced OC-genesis.

### 3.4. CV Stimulates OC Precursors via the PKC-δ Pathway

**Figure 3:**
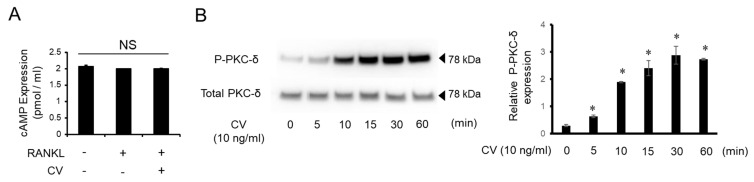
CV upregulates phosphorylation of PKC-δ in RANKL-stimulated OCs. (**A**) Amount of cAMP at 12 h after stimulation of CV (10 ng/mL). Results are shown as means ± SD. (**B**) Western blotting was performed on protein extracts from OC precursors stimulated with CV (10 ng/mL) for 0, 5, 10, 15, 30 and 60 min. Total amount of PKC-δ and p-PKC-δ was evaluated using the antibodies described in the Materials and Methods section. Densitometric analysis of p-PKC-δ signals is shown. The relative level of p-PKC-δ expression was normalized to total PKC-δ expression. All assays were performed in triplicate and the results were expressed as the mean ± SD. One representative picture band out of three experiments is shown. * *p* < 0.05.

To gain a more mechanistic insight into the CV-mediated upregulation of OC-genesis, PCR arrays were performed to explore the cell signaling pathway elicited by CV in the OC precursors and PKA/cAMP and/or PKC-δ pathways were indicated (data not shown). The level of cAMP in the OC precursors was not affected by the addition of CV (Figure 3A). On the other hand, according to W-blot, CV significantly promoted the phosphorylation of PKC-δ in the RANKL-stimulated OC precursors in a time-dependent manner (Figure 3B).

**Figure 4:**
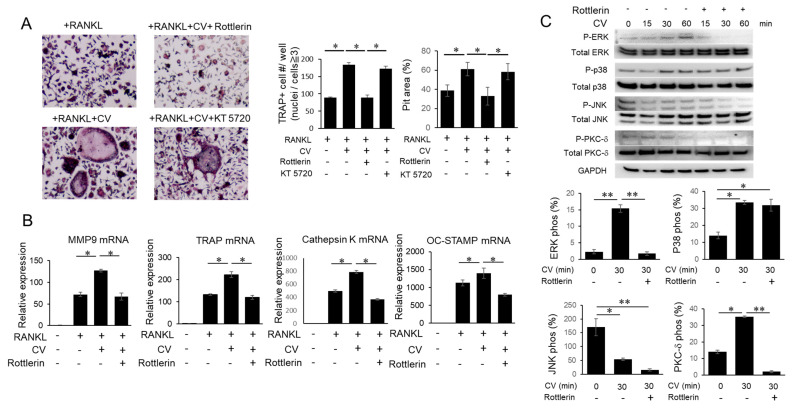
PKC-δ inhibitor can suppress CV-dependent elevated OC-genesis. Effect of PKC-δ inhibitor Rottlerin in comparison with PKA inhibitor KT5720 on the CV-dependent upregulation of OC-genesis was evaluated using mouse BM-derived OC precursors stimulated with M-CSF and RANKL. (**A**) OC precursors were stimulated with CV in the presence or absence of KT5720 (5 μM) or Rottlerin (5 μM) for 7 days with the addition of fresh medium every 3 days. TRAP staining and pit formation assays were performed. (**B**) Relative expression of MMP9, TRAP, Cathepsin K and OC-STAMP mRNAs in OC precursors was stimulated in the presence or absence of CV (10 ng/mL) with or without Rottlerin for 24 h. The relative fold changes of mRNA expression levels were determined by qPCR (normalized to GAPDH mRNA levels). (**C**) Western blotting was performed on protein extracts from OC precursors stimulated with CV (10 ng/mL) with or without Rottlerin for 0, 15, 30, and 60 min. Total p38^MAPK^, JNK, ERK and PKC-δ as well as phosphorylated counterparts were evaluated using the antibodies described in the Materials and Methods section. Densitometric analysis of phosphorylated p38^MAPK^, JNK, ERK and PKC-δ signals is shown. All assays were performed in triplicate. Data represent the mean ± SD of three independent experiments. Results were presented as the means ± SD. * *p* < 0.05; ** *p* < 0.01. Scale bar: 100 μm.

To determine if CV-elicited PKC-δ signaling could lead to the upregulation of OC-genesis, Rottlerin, a PKC-δ inhibitor, as well as KT5720, a PKA inhibitor, were applied to the RANKL-primed OC precursors. Rottlerin, but not KT5720, significantly inhibited the OC differentiation upregulated by CV (Figure 4A). Moreover, Rottlerin suppressed OC-related genes such as MMP9, TRAP, Cathepsin K and OC-STAMP mRNAs (Figure 4B). It has been reported that LPS-induced osteolysis is mediated by upregulated OC-genesis through the cell signaling of the PKC-δ/ERK-MAPK axis [52]. Therefore, we also investigated the effects of Rottlerin on the phosphorylation induced in three major MAPK molecules; namely, p38, ERK and JNK. Rottlerin inhibited CV-induced ERK phosphorylation, whereas it showed little or no effect on the phosphorylation of p38 and JNK (Figure 4C). These results indicated that the PKC-δ/ERK axis was involved in the CV-mediated upregulation of RANKL-induced OC-genesis.

### 3.5. Vimentin and CV Detected in Periodontitis Is Induced in Mice

**Figure 5:**
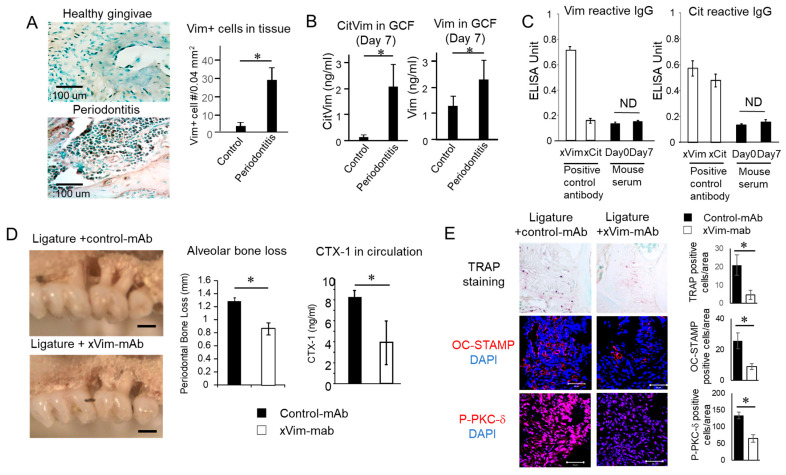
Possible pathogenic engagement of CV in the onset of periodontitis induced in mice. To monitor the expression pattern of CV and possible inhibitory effect of anti-vim mAb, a mouse model of ligature-induced periodontitis was employed (*n* = 5/group). (**A**) Expression of vimentin in periodontal lesion induced by ligation was visualized by immunohistochemistry. Infiltration of vimentin-positive cells was measured. (**B**) Expression of vimentin or CV in mice GCF was detected by ELISA, as described above. (**C**) Detection of anti-vimentin and anti-citrulline antibodies in murine serum. (**D**) Alveolar bone resorption around maxillary second molars induced by ligation for 7 days with injection of anti-vimentin mAb or control IgG was microscopically measured. CTX-1 concentration in serum of ligatured mice with injection of anti-vimentin mAb or control IgM was analyzed by ELISA. (**E**) Presence of TRAP-, OC-STAMP- or Phospho-PKC-δ-positive cells in the tissue section of mice induced with periodontitis is shown. Results are shown as means ± SD. * *p* < 0.05.

The possible expression of CV in periodontitis was evaluated in mice induced with periodontitis by the attachment of a ligature to the maxillary second molar. According to the immunohistochemistry of the decalcified alveolar bone isolated from mice euthanized on day 7, vimentin-positive mononuclear cells were detected in the tissue of mice induced with periodontitis, but not in the tissue collected from healthy control mice (Figure 5A). The level of vimentin and CV in the gingival crevicular fluid (GCF) collected on day 7 significantly increased in the group induced with periodontitis compared with that of the periodontally healthy control group (Figure 5B). Finally, an IgG antibody response to CV and vimentin was not detected in the serum samples collected from the mice on day 7 (Figure 5C).

### 3.6. mAb-Based Local Neutralization of Vimentin Suppressed Bone Resorption Is Induced in a Mouse Model of Periodontitis

Anti-vimentin mAb locally injected into the ligature-attached site during the induction of periodontitis suppressed alveolar bone resorption compared with a local injection of isotype-matched control mAb (Figure 5D). The level of bone resorption biomarker CTX-1 in the serum was also significantly suppressed by a local administration with anti-vimentin mAb (Figure 5D). Furthermore, TRAP-, OC-STAMP- and Phospho-PKC-δ-positive cells were decreased by an anti-vimentin mAb injection compared with isotype-matched control mAb in the mouse periodontitis lesions, suggesting that OC-mediated bone resorption induced by a ligature attachment was suppressed by anti-vimentin mAb (Figure 5E). 

## 4. Discussion

Our findings indicated that citrullinated vimentin (CV), but not intact vimentin (Vim), could promote osteoclast differentiation and function in conjunction with upregulating the expressions of osteoclast marker genes, including TRAP, MMP9, OC-STAMP and Cathepsin K, along with the activation of the cell signaling molecules PKC-δ and ERK. The mouse model of ligature-induced periodontitis demonstrated that the level of CV increased in the periodontal lesion, indicating that CV was, at least in part, engaged in the promotion of OC-genesis-mediated periodontal bone loss. However, the local administration of an anti-vimentin neutralizing monoclonal antibody (mAb) was able to suppress the periodontal bone loss induced by the attachment of a ligature, suggesting that CV is a potential target for the development of diagnostic and therapeutic approaches for periodontitis. 

Vimentin is a cytoskeletal intermediate filament expressed in mesenchymal cells as well as lymphocytes and neutrophils [53,54,55,56] and it plays a relevant role in cell motility. A unique feature of vimentin is derived from its susceptibility to citrullination by the PAD enzyme [57]. Indeed, CV is one of the major autoantigens for ACPA, which is used to decrease rheumatoid arthritis [58,59]. An anti-CV autoantibody isolated from human patients with rheumatoid arthritis was reported to induce OC-genesis in an ex vivo culture of human OC precursors derived from peripheral blood monocytes, whereas an anti-CV autoantibody was engaged in bone resorption in the tibia of Rag1-KO mice induced with rheumatoid arthritis [36,37]. However, we found that CV promoted osteoclast differentiation directly in the absence of an anti-CV autoantibody. Although the molecular mechanism underlying the anti-CV autoantibody-mediated promotion of OC-genesis in the context of rheumatoid arthritis remains unclear, it is feasible that CV may bind to the putative receptor expressed on OC precursors. 

That periodontitis is implicated as a risk factor for rheumatoid arthritis is supported by a high prevalence of periodontitis in rheumatoid arthritis patients [60,61]. The development of autoreactive B cells and the occurrence of ACPA in rheumatoid arthritis largely depends on the T cell response to HLA-restricted antigen presentation [62,63]. Indeed, autoreactive CD4+ effector T cells that react with CV have been observed in patients with rheumatoid arthritis [64,65], suggesting that the induction of an anti-CV autoantibody is mediated by a T cell response to CV. The association between periodontitis and the pathologic outcomes of rheumatoid arthritis is linked to the specific alleles in the highly polymorphic HLA-DRB1 locus [66]. Similar to dendritic cells and macrophages, OCs that are also derived from hematopoietic stem cells can function to present antigen to T cells in rheumatoid arthritis [67]. Similarly, through the possible presentation of CV through MHC class-II, OCs engaged in periodontitis lesions may be involved in anti-CV autoantibody production in a T cell-dependent manner in rheumatoid arthritis. In the present study, an elevation in periodontal bone loss in the ligature-attached mice was identified on day 7 when the anti-CV antibody was not detected in the blood serum (Figure 5). Therefore, an intriguing line of inquiry would be to address the possible induction of an anti-CV autoantibody in ligature-attached mice on day 8 when CV-reactive T cells may be activated. 

A PCR array (data not shown) was performed to delineate the cell signaling pathways induced by CV and the cAMP/PKA and PKC signaling pathways were depicted as the most likely candidates. Although CV did not induce cAMP in RANKL-primed OCs (Figure 3A), it did upregulate the phosphorylation of PKC-δ in RANKL-primed OCs (Figure 3B). Indeed, the engagement of PKC-δ in the induction of osteoclast differentiation has already been reported in several studies [52,68,69]. Kim et al. demonstrated that Rottlerin, a chemical inhibitor of PKC-δ, and shRNA targeting PKC-δ could suppress RANKL-primed osteoclast differentiation [69]. The PKC-δ inhibitor Rottlerin abrogated the CV-mediated upregulation of OC-genesis, whereas the PKA inhibitor KT5720 did not (Figure 4A). CV promoted the OC-genesis-related gene expression, including TRAP, MMP9, OC-STAMP and Cathepsin K mRNAs, all of which were suppressed by the addition of Rottlerin (Figure 4B), suggesting that the PKC-δ pathway plays a key role in the CV-mediated upregulation of OC-genesis. In Figure 3, CV was extracellularly applied to the OC precursors, not via an intracellular overexpression. According to a previous paper, RANKL did not induce PKC-δ phosphorylation in OCs [51]. Therefore, we speculated that PKC-δ phosphorylation was induced in the OCs in a manner specific to the extracellular application of CV or produced in an autocrine fashion. It followed that PKC-δ phosphorylation in OCs was mediated by CV that extracellularly acted on the OCs. To confirm the transduction of PKC-δ signaling, phosphorylated ERK (among the three major MAPKs) was found to be associated with the CV-mediated activation of PKC-δ (Figure 4C). It is true that the MAPK pathway also plays an important role in OC-genesis and bone resorption by OCs [70,71]. The engagement of PKC-δ and ERK-MAPK in CV-elicited cell signaling indicates that CV may be a ligand for receptor tyrosine kinases (RTKs) or G protein-coupled receptors (GPCRs) [72]. Accordingly, a future study will take up the detailed profiling of CV in relation to the initiation of cell signaling in OC precursors as well as the other cell signaling molecules downstream of PKC-δ and ERK-MAPK.

PAD2 and PAD4 expressed by various cell types of hematopoietic origin [73,74] are the main enzymes responsible for the citrullination of autoantigens in RA [73]. OCs constitutively express PAD4, whereas the expression of PAD2 is induced in a RANKL-dependent manner [36]. CV was detected in both the supernatant and cell lysate of RANKL-stimulated OC precursors (Figure 2A,B). Especially, the RANKL-dependent increase in citrullinated vimentin was suppressed by the presence of Cl-amidine (Figure 2A), suggesting that endogenously produced PADs were responsible for the citrullination of vimentin. Moreover, the anti-vimentin antibody significantly suppressed OC-genesis, pit formation and OC-related mRNA expression in RANKL-stimulated OC precursors (Figure 2D). A significantly elevated anti-vimentin antibody, but not the anti-MCV antibody, has been reported in patients with periodontitis compared with periodontally healthy control subjects [39]. It would be intriguing to study the association between the anti-vimentin antibody found in periodontitis patients and OC-genesis in the context of elevated vimentin in periodontitis lesions. The further upregulation of the CV-mediated promotion of OC-genesis by the anti-MCV antibody also remains an open question to be addressed in future studies. 

In the mouse model induced with periodontitis via a ligature attachment, vimentin-positive mononuclear cells were found in the alveolar bone (Figure 5A). Elevated CV was also detected in the GCF of mice induced with periodontitis compared with that of control non-ligatured mice (Figure 5B). These results indicated that mononuclear cells, including OC precursors, may be the cellular source of CV in mouse periodontitis lesions. Although PAD2 and PAD4 were expressed in inflamed gingiva isolated from human subjects [75,76], it is largely unknown whether mononuclear cells infiltrating the gingival tissue produce CV in humans. Despite the short induction period for the production of antibodies, neither the anti-vimentin antibody nor the anti-citrulline antibody was detected in the mouse serum collected on day 7 (Figure 5C). In other words, it is suggested that CV, not the anti-CV antibody, directly promotes OC-genesis in mouse periodontitis. On the other hand, a local injection of anti-vimentin mAb suppressed periodontal bone resorption in vivo (Figure 5D,E) instead of promoting it, suggesting that the neutralization of soluble vimentin may be a promising approach for the treatment of periodontitis.

## 5. Conclusions

In conclusion, the present study showed that CV directly promoted osteoclast differentiation via PKC-δ/ERK signaling and that CV was associated with the exacerbation of periodontal bone resorption induced in a mouse model of periodontitis, indicating that CV may serve as a candidate therapeutic molecule for future drug developments in the treatment and/or diagnosis of periodontitis.

## Data Availability

The data presented in this study are available on request from the corresponding author.
